# Supplementary material for: Microgeographic maladaptive performance and deme depression in response to roads and runoff
Source: PeerJ. 2013 Sep 17;1:e163. doi: 10.7717/peerj.163 (PMC3792186; doi:10.7717/peerj.163)
Supplement: Text S1 [file peerj-01-163-s001.docx]

**Supplemental Text**

Independent of the compounding effects of maladaptation and deme depression, roadside pools are harsh environments for aquatic stage amphibians. Regardless of deme, reciprocal transplant experiment results indicate that average survival in roadside pools was just 71% as compared to 89% in woodland pools (Posterior mean = -1.539; 95% HPD = -2.213– -0.898; *P_mcmc_* < 0.001), marking a 20% reduction in survival between the two environments. Though there was no evidence for a G x E interaction, or an effect of deme, growth and developmental rates were negatively affected by the roadside environment. As compared to the woodland environment, embryos raised to feeding stage in the roadside environment exhibited reduced rates of growth and development by 11.5% (Posterior mean = -0.007; 95% HPD = -0.0103 – -0.0029; *P_mcmc_* = 0.001) and 5% (Posterior mean = -0.006; 95% HPD = -0.0101 – -0.0025; *P_mcmc_* = 0.001), respectively (Fig. S1).

The consequences of road salt exposure on wood frog embryos and feeding stage larvae in controlled experimental environments are mixed as compared to exposure to natural sites. Acute exposure results indicate that increasing road salt concentration decreases survival, and that the roadside deme is more susceptible than the woodland deme to this effect (Fig. 3). Egg size had no effect on this outcome, nor did its presence as a covariate qualitatively alter the results. At the same time, chronic exposure indicates that road salt elevates the proportion of malformations for the roadside deme. However, there is weak support that the accrual of malformations at the medium road salt concentration might be influenced by embryo size, as the inclusion of this covariate shifted the contrast between demes closer to significance (table S5). Still, chronic exposure to road salt had no other negative consequences on embryonic performance variables (fig. S2). This is an especially striking comparison because the chronic exposure experiment was run concomitantly with the field transplant experiment, and for approximately the same length of time. It is also worth noting that larvae in the acute exposure experiment experienced reductions in survival at lower concentrations of road salt and over just a four day period, as compared to hatchlings in the chronic exposure experiment. A number of possibilities might explain this outcome. First, in a related study, exposure of wood frogs to comparable levels of road salt shows that larvae survive at lower rates than embryos([Karraker et al. 2008](#_ENREF_8)). Second, wood frogs entered into the acute exposure assay were first transferred from their natal water into a common environment of aged tap water and reared to feeding stage prior to the initiation of the experiment, and the intervening period of natal exposure may have had carryover effects on the larvae in the acute exposure. Wood frogs in the chronic exposure experiment, however, were transferred directly from their natal water into the chronic road salt treatments. Thus, embryos in the chronic exposure may have had the ability to acclimate as relatively undifferentiated embryos to the experimental road salt environment whereas larvae entered into the acute exposure may have experienced a shock in osmotic environment that exceeded their capacity for plastic response. This may be especially likely if we consider the developmental differences in the animals entering these two experiments. Specifically, the chronic exposure experiment was initiated with early cell division embryos surrounded by vitelline membranes whereas the acute exposure experiment was initiated with feeding stage larvae. Together, these results highlight the complexity of responses to wild conditions and suggest that singular based contaminant assays do not necessarily reflect the responses of wild populations.
