# Supplementary material for: Microgeographic maladaptive performance and deme depression in response to roads and runoff
Source: PeerJ. 2013 Sep 17;1:e163. doi: 10.7717/peerj.163 (PMC3792186; doi:10.7717/peerj.163)
Supplement: Supplemental Information S2 [file peerj-01-163-s002.docx]

**Supplemental Methods and Statistical Analyses**

*Characterizing roadside and woodland environments*

All water parameter measurements were taken 10 cm below the surface at the location of the deepest point in each pool. In roadside pools, specific conductance was also measured at the base of the water column because a strong halocline spans the water column. I therefore chose to analyze and report specific conductivity as the mean value taken from the top and bottom of each roadside pool. To estimate the influence of forest canopy cover, I captured leaf-off hemispherical photographs (on 1 April 2011) at each of five locations per pool (2 m from shore at each of the four cardinal compass points plus the approximate center of pool). I used HemiView software ([Delta-T Devices Ltd 1998-1999](#_ENREF_3)) to estimate global site factor (GSF)—a measure of solar radiation reaching the water surface ([see Halverson et al. 2003](#_ENREF_7)). GSF was calculated estimated for 10 Apr.

*Statistical analyses for reciprocal transplant, chronic, and acute responses*

All statistical analyses were conducted in R V. 2.15.0 ([R Development Core Team 2012](#_ENREF_9)). I used the *MCMCglmm* package ([Hadfield 2010](#_ENREF_6)) to analyze survival as a bivariate response of successes and failures, characterized by a binomial distribution (using the “multinomial2” family). This package uses a Bayesian approach to estimate parameters and generate inference. Specifically, unlike frequentist approaches—which infer the probability of the data obtained given the hypothesis posed—Bayesian approaches infer probability of the hypothesis given the data obtained ([Ellison 2004](#_ENREF_4)). *MCMCglmm* samples this so-called conditional probability or likelihood using Markov chain Monte Carlo methods. Inference is then generated from the sampled posterior distribution. Highest posterior density intervals (HPD) represent the interval over which the true mean is expected to occur with a specified confidence (typically 95%). *P_mcmc_* is reported as the smaller proportional value of the posterior estimates that are on either side of zero. Thus for the purpose of inference here, *P_mcmc_* is similar to a traditional *P* value. While interpretation of Bayseian-versus frequentist-derived analyses can differ, here inference is analogous. This is because I used weak, non-informative, parameter expanded priors. Thus the posterior distribution is influenced predominantly by the likelihood estimates.

To analyze growth and developmental rates, I used the R package *lme4* ([Bates et al. 2011](#_ENREF_2)). While *lme4* utilizes a frequentist framework, MCMC methods provides a more conservative method of inference due to the uncertainty associated with estimating degrees of freedom in hierarchical models. I therefore evaluated *lme*4 models using the function *pvals.fnc* in the *languageR* package ([Baayen 2011](#_ENREF_1)), which uses MCMC methods to compute *P_mcmc_* and HPD intervals. Growth and developmental rates were specified as exponential functions based on initial and final values of size and developmental stage, respectively, in relation to the number of days elapsed from stocking until the end of the experiment (e.g. [ln(final size)-ln(initial size)]/period). Initial size was defined as embryo diameter calculated from estimates of embryo area, while final size comprised hatchling snout-vent length (SVL). Developmental stages were assayed for embryos using the standard Gosner key ([Gosner 1960](#_ENREF_5)).

In all models, I specified deme and the experimental grow-out environment, along with their interaction (i.e. G x E), as fixed effects. A complimentary set of models was evaluated with egg size as a covariate. In the most parameterized reciprocal transplant model, the following random effects were included: pool pair, clutch pair, experimental block, and enclosure (i.e. a term for the observation level used to deal with overdispersion). In the least parameterized model, only pool pair was included. Similarly, for the chronic exposure experiment, the random effects pool pair and clutch pair were replaced with pool and clutch triad, respectively. In the acute exposure experiment, a random effect was included for exposure round. I evaluated model fit for each combination of random effects (Table S1-S3) and selected for inference the model with the lowest DIC or AIC values (for bivariate and univariate models, respectively). Models were run for 3,000,000 iterations, with a burnin period of 300,000, and a thinning interval of 500. Model performance was evaluated by examining traces of the posterior distributions of the location effects and covariance matrices. Models in which the interaction effect was found not to be significant were refit with only main effects (i.e. G + E), and the random effect structure was re-evaluated using the same procedure as above. Developmental and growth rate response variables were averaged by enclosure, while survival was measured as the number of successes and failures per enclosure. Estimates of parameters, HPD intervals, and *P_mcmc_* values are reported in Tables S4-S6.
