# Supplementary figures and images for: Microgeographic maladaptive performance and deme depression in response to roads and runoff
Source: PeerJ. 2013 Sep 17;1:e163. doi: 10.7717/peerj.163 (PMC3792186; doi:10.7717/peerj.163)

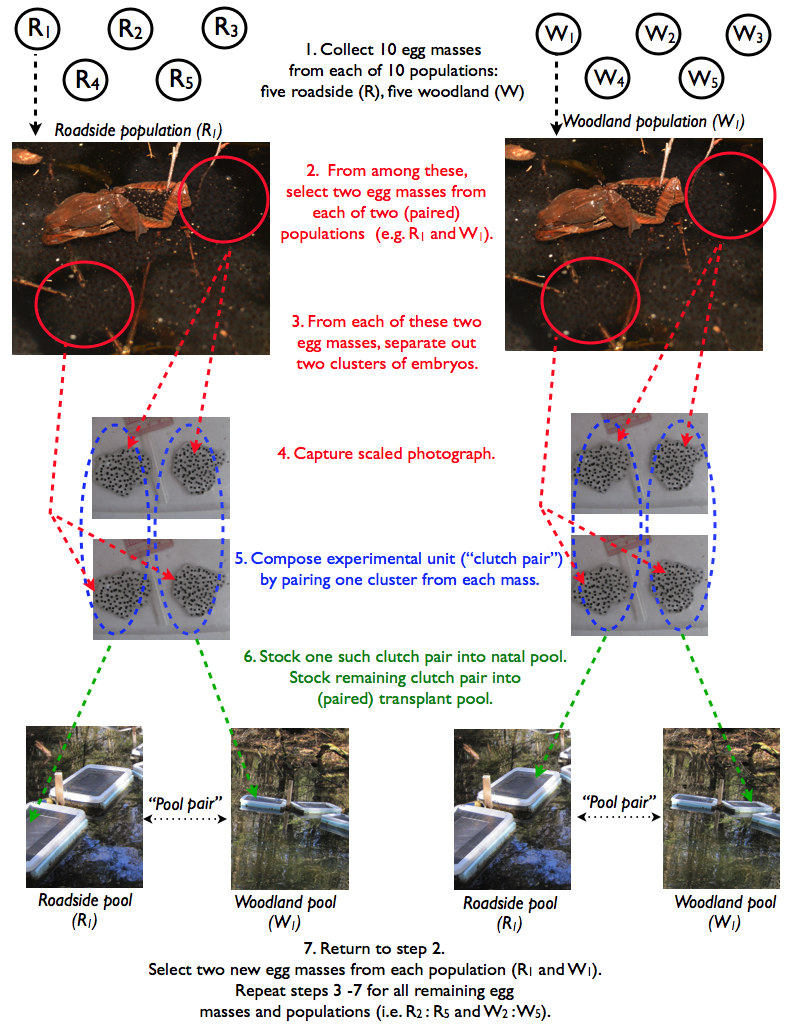

Supplement: Figure S1 — Procedural steps for stocking reciprocal transplant enclosures. The reciprocal transplant was conducted pairwise across ponds. Specifically, each of five roadside pools was paired with each of five nearby woodland pools; the transplantation of embryos occurred within each of these five pairs (see main text and Fig. 1). Text color corresponds to indicator arrows and ellipses. From top to bottom: 10 wood frog egg masses were collected from each of 10 pools (Step 1). A breeding pair of wood frogs is shown; red circles indicate the approximate extent of individual egg masses. “Clutch pairs” were formed by subsampling and combining ca. 50 embryos from each of two masses per population (Steps 2, 3, 5). Photographs were captured with a scale in view to allow estimation of embryo size (Step 4). Replicate cutch pairs were stocked into natal and transplant pools respectively; enclosures are shown. (Step 6). [file peerj-01-163-s003.png]
