# Supplementary material for: Microgeographic maladaptive performance and deme depression in response to roads and runoff
Source: PeerJ. 2013 Sep 17;1:e163. doi: 10.7717/peerj.163 (PMC3792186; doi:10.7717/peerj.163)
Supplement: Table S1 — Reciprocal transplant model selection results. A set of candidate models differing in random effects structure was composed for the analysis of each response variable. An observation level term (“obs”) was included to test and account for over-dispersion in the binomial model of survival. The model containing the fewest parameters with the lowest Akaike Information Criterion (AIC) score by a differential value of less than two was chosen for inference, and is indicated by a dagger (†). There was no significant genotype x environment (G x E) interaction in the model for survival, growth rate, or developmental rate. [file peerj-01-163-s007.docx]

**Table S1.** Reciprocal transplant model selection results. A set of candidate models differing in random effects structure was composed for the analysis of each response variable. An observation level term (“obs”) was included to test and account for over-dispersion in the binomial model of survival. The model containing the fewest parameters with the lowest Akaike Information Criterion (AIC) score by a differential value of less than two was chosen for inference, and is indicated by a dagger (†). There was no significant genotype x environment (G x E) interaction in the model for survival, growth rate, or developmental rate.

| ***Response variable ~ fixed effects*** | ***Random effects*** | ***AIC*** | |
| --- | --- | --- | --- |
|  |  | No embryo size covariate | With embryo size covariate |
| Survival ~ G + E | pair+clutch+block+obs  pair+clutch+block | 372.12†  408.94 | 373.50†  408.25 |
|  | pair+clutch+obs | 376.16 | 377.94 |
|  | pair+clutch  pair+block+obs | 891.44  382.54 | 893.40  384.46 |
|  | pair+block  pair+obs  pair | 902.61  380.54  1550.08 | 904.46  382.47  1551.91 |
| Growth rate ~ G + E | pair+clutch+block | -647.72 | -645.76 |
|  | pair+clutch | -625.47 | -623.52 |
|  | pair+block | -647.76† | -645.83† |
|  | pair | -627.47 | -625.52 |
| Developmental rate ~ G + E | pair+clutch+block | -696.40† | -694.42† |
|  | pair+clutch | -606.61 | -605.61 |
|  | pair+block | -620.95 | -622.66 |
|  | pair | -606.66 | -606.15 |
