# Supplementary material for: Microgeographic maladaptive performance and deme depression in response to roads and runoff
Source: PeerJ. 2013 Sep 17;1:e163. doi: 10.7717/peerj.163 (PMC3792186; doi:10.7717/peerj.163)
Supplement: Table S2 — Chronic exposure model selection results. Candidate models were composed for survival, growth, development, and malformations across the interaction of genotype (G) x environment (E); here environment refers to the three different road salt treatments. Model selection is described in caption for Table S1. An observation level term (“obs”) was included to test and account for over-dispersion in the binomial models. There was no significant G x E interaction in the model for survival, growth rate, or developmental rate. [file peerj-01-163-s008.docx]

**Table S2.** Chronic exposure model selection results. Candidate models were composed for survival, growth, development, and malformations across the interaction of genotype (G) x environment (E); here environment refers to the three different road salt treatments. Model selection is described in caption for Table S1. An observation level term (“obs”) was included to test and account for over-dispersion in the binomial models. There was no significant G x E interaction in the model for survival, growth rate, or developmental rate.

| **Response variable ~ fixed effects** | ***Random effects*** | **AIC (no embryo**  **size covariate)** | **AIC (with embryo size covariate)** |
| --- | --- | --- | --- |
| Survival ~ G + E | pool+clutch+block+obs | 258.71 | 256.30 |
|  | pool+clutch+block | 374.43 | 380.92 |
|  | pool+clutch+obs  pool+clutch  pool+block+obs  pool+block  pool+obs  pool | 256.83  376.91  257.50  436.30  255.50†  445.47 | 254.37  383.34  254.99  443.96  252.99†  452.56 |
| Growth rate ~ G + E | pool+clutch+block | -590.68 | -588.95 |
|  | pool+clutch | -592.68 | -590.95 |
|  | pool+block | -592.49 | -590.70 |
|  | pool | -594.34† | -592.54† |
| Developmental rate ~ G + E | pool+clutch+block | -483.28 | -481.47 |
|  | pool+clutch | -483.63† | -481.67† |
|  | pool+block | -477.63 | -476.17 |
|  | pool | -478.95 | -477.17 |
| Malformation ~ G x E | pool+clutch+block+obs | 177.84 | 177.84 |
|  | pool+clutch+block | 199.25 | 197.47 |
|  | pool+clutch+obs | 175.84 | 175.84 |
|  | pool+clutch | 205.57 | 204.60 |
|  | pool+block+obs | 175.84 | 175.84 |
|  | pool+block | 247.67 | 245.84 |
|  | pool+obs | 173.84† | 173.84† |
|  | pool | 253.52 | 251.56 |
