# Supplementary material for: Microgeographic maladaptive performance and deme depression in response to roads and runoff
Source: PeerJ. 2013 Sep 17;1:e163. doi: 10.7717/peerj.163 (PMC3792186; doi:10.7717/peerj.163)
Supplement: Table S3 — Acute exposure model selection results. Candidate models were composed for survival across the interaction of genotype (G) x environment (E); here environment refers to the five different road salt treatments. The model with the fewest parameters and lowest Akaike Information Criterion (AIC) score by a differential value of less than two was inferred, and is indicated by a dagger (†). Experiment block was nested within experiment round (“rnd”) because it was assumed that potential blocking effects would not be independent of experiment round. An observation level term (“obs”) was included to test and account for over-dispersion. [file peerj-01-163-s009.docx]

**Table S3.** Acute exposure model selection results. Candidate models were composed for survival across the interaction of genotype (G) x environment (E); here environment refers to the five different road salt treatments. The model with the fewest parameters and lowest Akaike Information Criterion (AIC) score by a differential value of less than two was inferred, and is indicated by a dagger (†). Experiment block was nested within experiment round (“rnd”) because it was assumed that potential blocking effects would not be independent of experiment round. An observation level term (“obs”) was included to test and account for over-dispersion.

| **Response variable ~ fixed effects** | ***Random effects*** | **AIC (no embryo**  **size covariate)** | **AIC (with embryo size covariate)** |
| --- | --- | --- | --- |
| Survival ~ G + E | pool+rnd+rnd:block+obs | 224.92 | 225.15 |
|  | pool+rnd+rnd:block | 257.06 | 257.39 |
|  | pool+rnd+obs  pool+rnd  pool+obs  pool | 222.93  259.55  222.32†  259.34 | 223.16  259.94  222.29†  259.51 |
