# Supplementary material for: Microgeographic maladaptive performance and deme depression in response to roads and runoff
Source: PeerJ. 2013 Sep 17;1:e163. doi: 10.7717/peerj.163 (PMC3792186; doi:10.7717/peerj.163)
Supplement: Table S4 — Parameter estimates, confidence intervals and p-values of field transplant models selected for inference. All models were composed with and without egg size as a covariate. [file peerj-01-163-s010.docx]

**Table S4.** Parameter estimates, confidence intervals and p-values of field transplant models selected for inference. All models were composed with and without egg size as a covariate.

| ***Model*** | ***Parameters*** | ***Estimate*** | ***Lower***  ***HPD*** | | ***Upper***  ***HPD*** | ***Pmcmc*** | |
| --- | --- | --- | --- | --- | --- | --- | --- |
|  |  | Without embryo size covariate /  With embryo size covariate | | | | | |
| Survival ~ G + E | Intercept | 3.114 /  4.047 | | 2.000 /  1.141 | 4.229 / 7.124 | | 0.003 / 0.008 |
|  | Deme | -0.964/ -0.907 | | -1.492 / -1.447 | -0.454 /  -0.352 | | 0.001 / <0.001 |
|  | Environment | -1.539 /  -1.537 | | -2.213 /  -2.117 | -0.898 /  -0.844 | | < 0.001 / <0.001 |
|  | Embryo size | NA /  -31.996 | | NA /  -140.129 | NA /  53.638 | | NA /  0.517 |
| Developmental rate ~ G + E | Intercept | 0.1507 / 0.1541 | | 0.1438 / 0.1320 | 0.1580/  0.1750 | | < 0.001 / < 0.001 |
|  | Deme | -0.0005 / -0.0004 | | -0.0043 / -0.0041 | 0.0030 / 0.0033 | | 0.755 / 0.856 |
|  | Environment | -0.0063 /  -0.0063 | | -0.0101 /  -0.0101 | -0.0025 /  -0.0026 | | 0.001 / 0.001 |
|  | Embryo size | NA /  -0.1166 | | NA /  -0.7898 | NA /  0.6009 | | NA /  0.734 |
| Growth rate ~ G + E | Intercept | 0.0555 / 0.0563 | | 0.0520 / 0.0396 | 0.0587 / 0.0733 | | < 0.001 /  < 0.001 |
|  | Deme | -0.0005 / -0.0005 | | -0.0038 / -0.0039 | 0.0028 / 0.0029 | | 0.753 / 0.772 |
|  | Environment | -0.0066 / -0.0066 | | -0.0103/ -0.0103 | -0.0029 /  -0.0029 | | 0.001/  0.001 |
|  | Embryo size | NA /  -0.0280 | | NA / -0.5967 | NA /  0.5289 | | NA /  0.9068 |
