# Supplementary material for: Microgeographic maladaptive performance and deme depression in response to roads and runoff
Source: PeerJ. 2013 Sep 17;1:e163. doi: 10.7717/peerj.163 (PMC3792186; doi:10.7717/peerj.163)
Supplement: Table S5 — Contrasts of G × E interaction on prevalence of malformations. Environment (E) refers to the three different road salt treatments. The model was composed with the original interaction effect of G × Ereformulated as one main effect (referred to as G × E†) comprising six levels (two demes X three treatments). Three contrasts were selected to infer responses between demes (R = roadside; W = woodland) within each treatment (L = low; M = medium; H = high). The remaining two orthogonal contrasts tested for differences between treatments irrespective of deme. [file peerj-01-163-s011.docx]

**Table S5.** Contrasts of G x E interaction on prevalence of malformations. Environment (E) refers to the three different road salt treatments. The model was composed with the original interaction effect of G x E reformulated as one main effect (referred to as G x E^†^) comprising six levels (two demes X three treatments). Three contrasts were selected to infer responses between demes (R = roadside; W = woodland) within each treatment (L = low; M = medium; H = high). The remaining two orthogonal contrasts tested for differences between treatments irrespective of deme.

| **Model** | ***Coefficient*** | ***Posterior mean*** | ***Lower HPD*** | ***Upper HPD*** | | ***Pmcmc*** |
| --- | --- | --- | --- | --- | --- | --- |
|  |  | Without embryo size covariate /  With embryo size covariate | | | | |
| Malformation ~ G x E^†^ | Intercept | -2.249 /  -3.588 | -2.454 /  -5.732 | -2.023/  -1.364 | < 0.001 /  0.001 | |
|  | RL vs. WL | 0.0009 /  0.027 | -0.417 /  -0.396 | 4.155 /  0.419 | 0.998 /  0.906 | |
|  | RM vs. WM | 0.263 /  0.296 | -0.043 /  -0.006 | 0.562 /  0.610 | 0.098 /  0.061 | |
|  | RH vs. WH | -0.364 /  -0.334 | -0.625 /  -0.607 | -0.081 /  -0.063 | 0.007 /  0.013 | |
|  | M vs. L | 3.337 /  3.318 | 2.756 /  2.780 | 3.875 /  3.908 | < 0.001 /  < 0.001 | |
|  | M vs. H | -3.653 /  -3.656 | -4.106 /  -4.077 | -3.212 /  -3.203 | < 0.001 /  < 0.001 | |
|  | Embryo size | NA /  44.720 | NA /  -30.841 | NA /  114.389 | NA /  0.222 | |
