# Supplementary material for: Microgeographic maladaptive performance and deme depression in response to roads and runoff
Source: PeerJ. 2013 Sep 17;1:e163. doi: 10.7717/peerj.163 (PMC3792186; doi:10.7717/peerj.163)
Supplement: Table S6 — Parameter estimates, confidence intervals and p-values of acute exposure model selected for inference. The interaction term was marginally significant and so was retained in the model. Embryo size could not be included in the model because clutch groupings were not separately maintained in the experiment. [file peerj-01-163-s012.docx]

**Table S6.** Parameter estimates, confidence intervals and p-values of acute exposure model selected for inference. The interaction term was marginally significant and so was retained in the model. Embryo size could not be included in the model because clutch groupings were not separately maintained in the experiment.

| ***Model*** | ***Parameters*** | ***Estimate*** | ***Lower***  ***HPD*** | | ***Upper HPD*** | ***Pmcmc*** | |
| --- | --- | --- | --- | --- | --- | --- | --- |
| Survival ~ G X E | Intercept | 17.497 | | 12.241 | 23.194 | | <0.001 |
|  | Deme | -7.569 | | -14.517 | -0.508 | | 0.038 |
|  | Concentration | -2.422 | | -2.994 | -1.868 | | <0.001 |
|  | Deme X Concentration | 0.637 | | -0.042 | 1.264 | | 0.059 |
