# Supplementary material for: Microgeographic maladaptive performance and deme depression in response to roads and runoff
Source: PeerJ. 2013 Sep 17;1:e163. doi: 10.7717/peerj.163 (PMC3792186; doi:10.7717/peerj.163)
Supplement: Table S7 — Parameter estimates of environmental variables. Estimates were obtained from univariate ANOVAs on each environmental variable following an overall significant MANOVA (Pillai’s Trace = 1.00, df = 8, 1, P = 0.016). For each variable, estimates are given for both the intercept and the pool type, the latter showing the effect with respect to roadside pools designated as the reference level. Specific conductance and pool area were log-transformed to improve normality. The multivariate analysis was conducted in R using the function manova, while univariate analyses were conducted using the function lm, both of which are in the Base Package. [file peerj-01-163-s013.docx]

**Table S7. Parameter estimates of environmental variables.** Estimates were obtained from univariate ANOVAs on each environmental variable following an overall significant MANOVA (Pillai’s Trace = 1.00, df = 8, 1, *P* = 0.016). For each variable, estimates are given for both the intercept and the pool type, the latter showing the effect with respect to roadside pools designated as the reference level. Specific conductance and pool area were log-transformed to improve normality. The multivariate analysis was conducted in R using the function *manova*, while univariate analyses were conducted using the function *lm*, both of which are in the Base Package.

| ***Environmental Variable*** | ***Parameters*** | ***Estimate*** | ***Standard Error*** | ***T*** | ***P*** |
| --- | --- | --- | --- | --- | --- |
| Specific conductance | Intercept | 1.534 | 0.053 | 29.07 | < 0.001 |
|  | Type | 1.385 | 0.075 | 18.55 | < 0.001 |
| Dissolved oxygen | Intercept | 2.632 | 0.082 | 32.10 | < 0.001 |
|  | Type | -0.254 | 0.116 | -2.19 | 0.060 |
| Depth | Intercept | 77.00 | 13.14 | 5.73 | < 0.001 |
|  | Type | 10.60 | 19.01 | 0.56 | 0.592 |
| GSF | Intercept | 0.620 | 0.060 | 10.36 | < 0.001 |
|  | Type | -0.064 | 0.085 | -0.755 | 0.472 |
| Area | Intercept | 3.013 | 0.155 | 19.50 | < 0.001 |
|  | Type | 0.113 | 0.219 | 0.516 | 0.620 |
| pH | Intercept | 5.700 | 0.200 | 28.57 | < 0.001 |
|  | Type | -0.140 | 0.282 | -0.50 | 0.633 |
| Temperature | Intercept | 11.51 | 0.840 | 13.71 | < 0.001 |
|  | Type | -0.568 | 1.187 | -0.48 | 0.645 |
